# Mitogen- and Stress-Activated Kinase 1 (MSK1) Regulates Cigarette Smoke-Induced Histone Modifications on NF-κB-dependent Genes

**DOI:** 10.1371/journal.pone.0031378

**Published:** 2012-02-01

**Authors:** Isaac K. Sundar, Sangwoon Chung, Jae-woong Hwang, John D. Lapek, Michael Bulger, Alan E. Friedman, Hongwei Yao, James R. Davie, Irfan Rahman

**Affiliations:** 1 Department of Environmental Medicine, Lung Biology and Disease Program, University of Rochester Medical Center, Rochester, New York, United States of America; 2 Department of Pediatrics, University of Rochester Medical Center, Rochester, New York, United States of America; 3 Manitoba Institute of Cell Biology, University of Manitoba, Winnipeg, Manitoba, Canada; Johns Hopkins School of Medicine, United States of America

## Abstract

Cigarette smoke (CS) causes sustained lung inflammation, which is an important event in the pathogenesis of chronic obstructive pulmonary disease (COPD). We have previously reported that IKKα (I kappaB kinase alpha) plays a key role in CS-induced pro-inflammatory gene transcription by chromatin modifications; however, the underlying role of downstream signaling kinase is not known. Mitogen- and stress-activated kinase 1 (MSK1) serves as a specific downstream NF-κB RelA/p65 kinase, mediating transcriptional activation of NF-κB-dependent pro-inflammatory genes. The role of MSK1 in nuclear signaling and chromatin modifications is not known, particularly in response to environmental stimuli. We hypothesized that MSK1 regulates chromatin modifications of pro-inflammatory gene promoters in response to CS. Here, we report that CS extract activates MSK1 in human lung epithelial (H292 and BEAS-2B) cell lines, human primary small airway epithelial cells (SAEC), and in mouse lung, resulting in phosphorylation of nuclear MSK1 (Thr581), phospho-acetylation of RelA/p65 at Ser276 and Lys310 respectively. This event was associated with phospho-acetylation of histone H3 (Ser10/Lys9) and acetylation of histone H4 (Lys12). MSK1 N- and C-terminal kinase-dead mutants, MSK1 siRNA-mediated knock-down in transiently transfected H292 cells, and MSK1 stable knock-down mouse embryonic fibroblasts significantly reduced CS extract-induced MSK1, NF-κB RelA/p65 activation, and posttranslational modifications of histones. CS extract/CS promotes the direct interaction of MSK1 with RelA/p65 and p300 in epithelial cells and in mouse lung. Furthermore, CS-mediated recruitment of MSK1 and its substrates to the promoters of NF-κB-dependent pro-inflammatory genes leads to transcriptional activation, as determined by chromatin immunoprecipitation. Thus, MSK1 is an important downstream kinase involved in CS-induced NF-κB activation and chromatin modifications, which have implications in pathogenesis of COPD.

## Introduction

Cigarette smoke (CS) contains more than 10^14–16^ free radicals/oxidants per puff and is composed of ∼4700 chemical compounds, including a major aldehyde, acrolein. CS mediates pro-inflammatory effects by carbonyl and oxidative stress in the lung via the generation of reactive oxygen species (ROS) and aldehydes, as well as through endogenous generation of ROS from inflammatory/structural cells [1], [2]. Pulmonary inflammatory response due to the infiltration of macrophages and neutrophils in the interstitium plays a central role in the pathogenesis of chronic obstructive pulmonary disease (COPD) [3], [4]. Furthermore, chronic CS exposure to mice leads to increased lung inflammatory response and airspace enlargement, which are characteristics of COPD/pulmonary emphysema [2], [5]. Inflammatory cells release numerous mediators that can cause airway constriction and remodeling. These cells also produce proteases (elastase, cathepsins, granzymes, and MMPs) that can cause destruction of lung parenchyma leading to airspace enlargement [2], [4], [5]. Changes in the levels of pro-inflammatory mediators are associated with activation of specific transcription factors, such as nuclear factor-kappaB (NF-κB), in the lung [1], [3], [6]. Site-specific posttranslational modifications, such as phosphorylation and acetylation of RelA/p65 (a subunit of NF-κB), play an essential role in NF-κB activation and CS-mediated lung inflammation [7], [8].

Mitogen- and stress-activated protein kinase 1 (MSK1) plays an important role in transcriptional activation of NF-κB-dependent pro-inflammatory genes, but the mechanism by which it functions, including the potential roles in chromatin modifications in response to environmental stimuli, is not known. MSK1 is a nuclear kinase that in a stimulus-dependent fashion associates with RelA/p65 and phosphorylates RelA/p65 at Ser276. Hence, MSK1 serves as a specific NF-κB RelA/p65 kinase, which may have an important signaling intermediate for chromatin modifications [9]. We have shown that CS-derived oxidants and aldehydes cause chromatin modifications on pro-inflammatory gene promoters, leading to sustained lung inflammation in smokers and patients with COPD [1], [2], [10], but the underlying mechanism for chromatin modifications, leading to histone acetylation on promoters of pro-inflammatory genes remains poorly understood. We hypothesized that MSK1 mediates chromatin modifications and forms multi-nuclear complexes with RelA/p65 and p300 in response to CS on promoters of pro-inflammatory genes.

MSK1 mediates the growth-factor, cellular and mitogenic stress-induced activation of transcription factors and chromatin proteins, such as cAMP-response-element-binding (CREB), activating transcription factor 1 (ATF1), NF-κB, and the chromatin protein histone H3, and high-mobility group protein (HMG) 14 [11], [12], [13]. In humans, phosphorylation at Thr581 located within the C-terminal kinase domain is essential for activation of MSK1 [13]. Hence, it is possible that environmental stimuli, such as cigarette smoke may activate MSK1 via phosphorylation of Thr581. Phosphorylation of NF-κB at Ser276 promotes the recruitment of the transcriptional coactivator p300/CBP, which in turn acetylates both RelA/p65 and histones at NF-κB bound promoters [7], [14]. However, the role of MSK1 in transcriptional activation of NF-κB RelA/p65 and histone modifications by CS is not known. Here, we report that CS induces histone modifications via MSK1 and phospho-acetylation of RelA/p65, followed by recruitment of MSK1 and its substrates to the promoters of pro-inflammatory genes, thereby increasing transcription of genes encoding pro-inflammatory mediators. Our data reveals the mechanism by which MSK1 is involved in CS-mediated NF-κB activation and chromatin modifications in human bronchial epithelial cells (H292 and BEAS-2B), human primary small airway epithelial cells (SAEC), and in lungs of mice exposed to CS. We report for the first time that CS-mediated activation of MSK1 forms a complex with NF-κB RelA/p65 and p300, which plays a key role in histone modifications of NF-κB-dependent pro-inflammatory gene promoters.

## Materials and Methods

### Ethics statement

All experimental protocols were performed in accordance with the standards established by the United States Animal Welfare Act, as set forth by the National Institutes of Health guidelines. The research protocol for these studies was approved by the University of Rochester Committee on Animal Research.

### Materials

Unless otherwise stated, all biochemical reagents used in this study were purchased from Sigma Chemicals (St. Louis, MO, USA). Penicillin-streptomycin, L-glutamine and RPMI-1640 were obtained from Gibco BRL (Grand Island, NY). Fetal bovine serum (FBS) was obtained from HyClone Laboratories (Logan, UT). Dulbecco's modified Eagle's medium-Ham's F12 50∶50 mixture (DMEM-F12) was purchased from Mediatech (Manassas, VA). Amphotericin B was purchased from Lonza (Walkersville, MD). MSK1, p-S276 (RelA/p65), RelA/p65, and Lamin B antibodies were obtained from Santa Cruz Biotechnology (Santa Cruz, CA). Anti-Flag M2, and â-actin antibodies were obtained from Sigma. p-MSK1 (Thr581), Ac-K310 RelA/p65, p-Ac-(Ser10/Lys9) histone H3, Ac-(Lys12) histone H4, histone H3 and histone H4 antibodies were obtained from Cell Signaling (Danvers, MA).

### Cell culture

Human bronchial epithelial cells (H292) were obtained from the American Type Culture Collection (ATCC) (Manassas, VA). H292 cells were grown in RPMI-1640 supplemented with 10% FBS, 2 mM L-glutamine, 100 U/ml penicillin and 100 µg/ml streptomycin. Human bronchial epithelial cells (BEAS-2B) [15] obtained from ATCC were grown in DMEM-F12 50∶50 mixture (DMEM-F12; Mediatech, Manassas, VA USA) supplemented with 5% FBS, 15 mM HEPES, 100 µg/ml penicillin, and 100 U/ml streptomycin. Human small airway epithelial cells (SAECs) derived from a single healthy donor were purchased from Lonza (formerly Cambrex, Walkersville, MD, USA) along with growth medium [Small Airway Epithelial Cell Growth Medium (SAGM)] bullet kit™ supplemented with other factors as provided by the supplier. The cells were cultured at 37°C in a humidified atmosphere containing 7.5% CO_2_. Mouse embryonic fibroblast (MEF) 10T1/2 (wild-type) and stable MSK1 knockdown 10T1/2 cells were grown in á-MEM medium supplemented with 10% FBS, 100 U/ml penicillin, 100 µg/ml streptomycin and 250 ng/ml amphotericin B. Puromycin was used as selection agent (8 µg/ml) for the growth and maintenance of stable MSK1 knockdown cells [16]. MEF were cultured at 37°C in a humidified atmosphere containing 5% CO_2_.

### Recombinant cDNA constructs, siRNA, and stable MSK1 knock-out mouse embryonic fibroblasts

pCMV-FLAG-MSK1 wild-type, pCMV-FLAG-MSK1 D195A (N-terminal kinase-dead mutant denoted as ND mutant) and pCMV-FLAG-MSK1 D565A (C-terminal kinase-dead mutant denoted as CD mutant) were kind gifts from Dr. Simon J Arthur (MRC Protein Phosphorylation Unit, School of Life Sciences, University of Dundee, Scotland, UK) [17]. Flag-p65 wild-type and Flag-p65 S276A (mutant) were kind gifts from Dr. James M. Samet (Center for Environmental Medicine Asthma and Lung Biology, University of North Carolina, Chapel Hill, NC) [18]. Knock-down of MSK1 expression in H292 cells was achieved by transfection of small interfering RNAs (siRNA). The siRNAs of human MSK1 (On-TARGET-Plus SMART Pool human RPS6KA5; Cat# L-004665-00) and silencer-negative control siRNA (Non-Targeting siRNA; Cat# D-001210-01-00) were purchased from Dharmacon (Thermo Scientific, Lafayette, CO). Transfection of siRNA was performed using the DharmaFECT 2 transfection reagent according to the manufacturer's instructions (Thermo Scientific, Waltham, MA). Mouse fibroblast 10T1/2 cells (wild-type) contained empty GIPZ lentiviral vector and MSK1 stable knock-down mouse fibroblast contained GIPZ lentiviral shRNAmir clones for mouse MSK1. These cells were kindly provided by Dr. James R. Davie (Manitoba Institute of Cell Biology, University of Manitoba, Manitoba, Canada) [16].

### Transfection

Recombinant plasmids used in the study were transiently transfected into cells grown in 6-well culture plates (7.0×10^5^ cells) or 100 mm dishes (4×10^6^ cells) in a total volume of 2 ml or 7 ml RPMI-1640 containing 10% FBS without antibiotics overnight, respectively. The cells were transiently transfected with recombinant wild type and mutant vectors (4–8 µg) using Lipofectamine 2000 (Invitrogen, CA) according to the manufacturer's instructions. Transfection efficiency in case of plasmid transfections was >70%. H292 cells were treated with CSE (1% or 2%) for 1 hr 24 hrs after transfection and the whole cell lysate was used for Western blot analysis.

### Preparation of cigarette smoke extract

Research grade cigarettes 3R4F were obtained from the Kentucky Tobacco Research and Development Center at the University of Kentucky (Lexington, KY). Ten percent CSE was prepared by bubbling smoke from one 3R4F research-grade cigarette into 10 ml of culture medium at a rate of one cigarette/min, as described previously [10], [19], using a modification of the method as described [20]. The pH of CSE was adjusted to 7.4 and then sterile-filtered through a 0.45 µm filter (25-mm Acrodisc; Pall Corporation, Ann Arbor, MI, USA). CSE preparation was standardized by measuring the absorbance at 320 nm (OD = 1.00±0.05). The spectral variations observed between different CSE preparations at 320 nm were minimal. CSE (10%), contains up to 394 µM acrolein [21] equivalent to 39.4 µM in 1% CSE. For each experiment, freshly prepared CSE was diluted with culture media without FBS within 10 min of preparation before use. Control medium was prepared by bubbling air through 10 ml of culture medium without FBS, adjusting pH to 7.4, and sterile filtering as described above. Acrolein and acetaldehyde are the main aldehyde components present in CS, and one cigarette yields ∼500 µg of acrolein and ∼1000 µg of acetaldehyde depending on cigarette type and determination methods [21], [22], [23], [24]. Moreover, we used CSE (0.1%–2%) to treat cells, which is equivalent to ∼10–40 µM aldehydes present in CS [22].

### Mouse cigarette smoke exposure

Wild-type (WT) mice of genetic background C57BL/6J (Jackson Laboratory, Bar Harbor, ME, USA) were bred and maintained under pathogen-free conditions with a 12 h light/dark cycle in the vivarium facility of the University of Rochester. Adult C57BL/6J mice were exposed to CS using research grade cigarettes (3R4F) according to the Federal Trade Commission protocol (1 puff/min of 2 s duration and 35 ml volume) using a Baumgartner-Jaeger CSM2072i automatic CS generating machine (CH Technologies, Westwood, NJ). Mainstream CS was diluted with filtered air and directed into the exposure chamber. The smoke exposure [total particulate matter (TPM) in per cubic meter of air] was monitored in real-time with a MicroDust Pro-aerosol monitor (Casella CEL, Bedford, UK) and verified daily by gravimetric sampling. The smoke concentration was set at a value of ∼300 mg/m^3^ TPM by adjusting the flow rate of the diluted medical air, and the level of carbon monoxide in the chamber was 350 ppm [1], [2], [10]. One cigarette yields ∼500 µg of acrolein and ∼1000 µg of acetaldehyde depending on cigarette type and determination methods [21], [22], [23], [24]. Hence, CS at a dose of ∼300 mg/m^3^ TPM (corresponding to human consumption of 1–1.5 packs per day) [25] was used in our study. Mice (*n* = 4 per group) received two 1 h exposures (1 h apart) daily for three consecutive days, and were sacrificed at 24 h post-final exposure. Control mice were exposed to filtered air in an identical chamber according to the same protocol as described for CS exposure. Mice were anesthetized by an intraperitoneal injection of pentobarbital sodium (100 mg/kg; Abbott Laboratories, Abbott Park, IL), and then sacrificed by exsanguination 24 h after last exposure. The lungs were removed *en bloc* and frozen for immunoblotting and immunoprecipitation analyses.

### Cell treatments

Human epithelial cells H292, BEAS-2B and SAEC cells (7×10^5^ and 4×10^6^) were grown in 6-well or 100 mm dishes to ∼80–90% confluency in respective cell culture medium with 0.5% FBS. The cells were treated with CSE (0.1%, 0.5%, 1% and 2%) for 1 h at 37°C with 7.5% CO_2_. At the end of treatment, the cells were washed with cold sterile Ca^2+^/Mg^2+^-free PBS and lysed using the RIPA buffer supplemented with a protease inhibitor cocktail (leupeptin, aprotinin, pepstatin, and PMSF) for whole cell extract preparation. For CSE treatment, mouse embryonic fibroblast (10T1/2 wild-type and MSK1 stable knockdown) cells were grown ∼70–80% confluent. 10T1/2 cells were serum starved for 24 h in α-MEM medium supplemented with 0.5% FBS and treated with CSE (1%) for 1 hr at 37°C with 5% CO_2_.

### Nuclear protein isolation and acid extraction of histone proteins

For nuclear extracts, cells were lysed in buffer A (10 mM HEPES [pH 7.9], 10 mM KCl, 0.1 mM EDTA, 0.1 mM EGTA, 1 mM DTT, 0.5 mM PMSF). After 15 min, Nonidet P-40 was added to a final concentration of 0.6% and vortexed for 15 sec. Samples were centrifuged for collection of the supernantants containing cytosolic proteins. The nuclear pellets were resuspended in buffer B (20 mM HEPES [pH 7.9], 0.4 M NaCl, 1 mM EDTA, 1 mM EGTA, 1 mM DTT, 1 mM PMSF). After 30 min at 4°C lysates were centrifuged and supernatants containing the nuclear proteins were stored at −80°C. The final pellet obtained after nuclear extraction was used for preparation of histones by acid-extraction. To the nuclear pellet, 150 µl of acid extraction buffer containing 0.2 N HCl and 0.36 N H_2_SO_4_ was added. The mixture was rotated on a rocker at 4°C for 6–18 hrs. If necessary, the pellet was sonicated for 2 sec on ice and centrifuged at 14,000 g for 10 min at 4°C. Then the supernatant containing acid extracted histones was transferred to a fresh tube and mixed with 1.1 ml of ice-cold acetone to precipitate histones. The tubes were incubated in −20°C overnight, then centrifuged at 14,000 g for 10 min at 4°C. The pellet was washed again with ice-cold acetone and centrifuged to remove the acid. The pellet was then air dried and dissolved in sterile distilled water for protein quantification using bicinchoninic acid (BCA) kit (Pierce, Rockford, IL, USA) followed by immunoblot analysis.

### Protein extraction from lung tissue

Mice were injected with sodium pentobarbital (100 mg/kg body weight, i.p.; Abbott Laboratories, Chevy Chase, MD, USA) and sacrificed by exsanguination. The lungs were removed *en bloc*, and the left lungs were lavaged 3 times with 0.5 ml of 0.9% NaCl, while the right lung lobe was frozen for immunoblot analysis. One hundred milligrams of right lung lobe was mechanically homogenized in 0.5 ml of ice-cold RIPA buffer supplemented with a protease inhibitor cocktail (leupeptin, aprotinin, pepstatin, and PMSF), and then placed on ice for 45 min to allow for total cell lysis to occur. The homogenate was centrifuged at 13,000 g in a bench-top centrifuge for 25 min at 4°C for removal of cellular debris. The supernatant was then transferred to a fresh 1.7-ml eppendorf tube and used as whole lysate.

### Immunoblot analysis

Protein levels were measured using the BCA kit. Twenty micrograms of whole cell extracts or nuclear extracts of epithelial cells (BEAS-2B, H292 or SAEC) or 20 µg of protein from whole lung homogenate were prepared as described above, subjected to electrophoresis on 6.5% (for whole cell extracts or nuclear extracts) or 14% (for acid-extracted histone proteins) SDS-PAGE gels and transferred onto nitrocellulose membranes (Amersham, Arlington Heights, IL, USA). The nitrocellulose membrane was blocked with 5% BSA and subsequently incubated overnight at 4°C with specific primary antibodies (1∶1000 dilution). After 3 washing steps (10 min each), the levels of protein were detected by probing with specific secondary anti-rabbit -mouse or -goat antibody (1∶20,000 dilution) linked to horseradish peroxidase (Dako, Santa Barbara, CA, USA) for 1 h, and bound complexes detected using the enhanced chemiluminescence method (ECL; Perkin-Elmer, Waltham, MA).

### Immunoprecipitation

Transfected H292 cells were lysed in 100 µl lysis buffer and 250–500 µg cell lysate was immunoprecipitated overnight with 40 µl EZview Red anti-flag M2 affinity gel (Sigma). Beads were washed with Tris-buffered saline and immuno-complexes separated by SDS–PAGE. For immunoprecipitation of RelA/p65, MSK1, and p300, 500–1000 µg cell lysates (nuclear or whole cell extracts) were incubated with 4 µg of specific antibodies overnight at 4°C, then immunoprecipitated with protein A/G Agarose beads (Santa Cruz Biotech) for 1 h at 4°C. Beads were washed with lysis buffer, boiled for 5 min in 2× sample buffer and immuno-complexes separated on SDS–PAGE. Proteins were transferred overnight onto nitrocellulose membranes (Amersham) and probed with respective primary antibodies overnight. After 3 washing steps (10 min each), primary antibodies were detected using the specific secondary anti-rabbit or -mouse antibodies for 1 h at room temperature, and the bound complexes detected using the enhanced chemiluminescence method (PerkinElmer).

### Immunofluorescence

H292, SAEC, and mouse fibroblast 10T1/2 (wild-type) and MSK1 stable knock-down mouse fibroblast cells were grown on 8-well chamber slides (1×10^4^ cells/well), treated with CSE (1%) for 1 h and fixed in 4% paraformaldehyde for 10 min. The cells were then permeabilized for 10 min in 0.4% Triton X-100 in PBS, and blocked for 1 h using 10% normal goat serum with 0.4% Triton X-100. The samples were incubated with specific primary antibody in a humidified chamber overnight. The primary antibody was detected with Alexa Fluor 594 goat anti-rabbit or Alexa Flour 555 donkey anti-mouse secondary antibody (Invitrogen, Carlsbad, CA, USA). Nuclei were stained with 1 µg/ml Hoechst 33342 for 1 min. Samples without primary antibodies were used as negative controls. The coverslips were mounted onto the slides using VectaShield (Vector Laboratories, Burlingame, CA, USA) and viewed under a Nikon TE2000-E microscope (Nikon, Tokyo, Japan).

### Chromatin immunoprecipitation (ChIP) assay

ChIP assay was performed using the Imprint ChIP kit (Sigma) according to the manufacturer's instructions. Briefly, H292 (1.6×10^6^) cells were cross-linked with 1% formaldehyde for 10 min and then quenched by adding 1.25 M glycine. Cells were centrifuged at ∼180 g for 5 min and washed 3 times with 10 ml ice-cold PBS by centrifugation. Cell nuclei were isolated and the pellet resuspended in shearing buffer, and then sonicated 7 times with 7 sec pulses on ice. Equal amounts of sonicated chromatin were immunoprecipitated with 1 µg of specific antibodies [MSK1, phospho-RelA/p65 (S276), acetylated histone H3 (Lys9) and histone H4 (Lys12), mouse IgG as negative control and RNA polymerase II as positive control]. Reversal of crosslinks and DNA purification were performed on immunoprecipitated samples and on input DNA as described in the kit. PCR amplification was performed using a PTC-200 DNA engine (M.J. Research, Waltham, MA) employing the following conditions: 94°C for 3 min; 32–35 cycles at 94°C for 45 sec, 60°C for 1 min, and 72°C for 1 min; and final extension at 72°C for 10 min. Human pro-inflammatory gene promoter sequence specific primer sequences and amplification product sizes (bp) are listed in Table 1. The PCR products were analyzed on a 1.5% agarose gel.

**Table 1:** Human pro-inflammatory gene promoters primer sequences used in chromatin immunoprecipitation assay.

Gene	Primer Sequence	PCR product (bp)
IL-6	Sense 5′-TTGCGATGCTAAAGGACG-3′Antisense 5′TGTGGAGAAGGAGTTCATAGC-3′	257
IL-8	Sense 5′-GTTGTAGTATGCCCCTAAGAG-3′Antisense 5′-CTCAGGGCAAAC CTGAGTCATC-3′	407
COX-2	Sense 5′-CAAGGCGATCAGTCCAGAAC-3′Antisense 5′-GGTAGGCTTTGCTGTCTGAG-3′	464

### Statistical analysis

Results are shown as mean ± SEM. Statistical analysis of significance was calculated using one-way analysis of variance (ANOVA) followed by Tukey's *post hoc* test for multigroup comparisons using the StatView software. Image J software (Version 1.41, National Institutes of Health, Bethesda, MD) was used for quantification of corrected total cell fluorescence (CTCF) by measuring cell fluorescence and densitometry analysis. *P*<0.05 was considered as significant, whereas *P*>0.05 was considered as non-significant.

## Results

### CS causes activation of MSK1 and its substrate NF-κB RelA/p65

**Figure 1:**
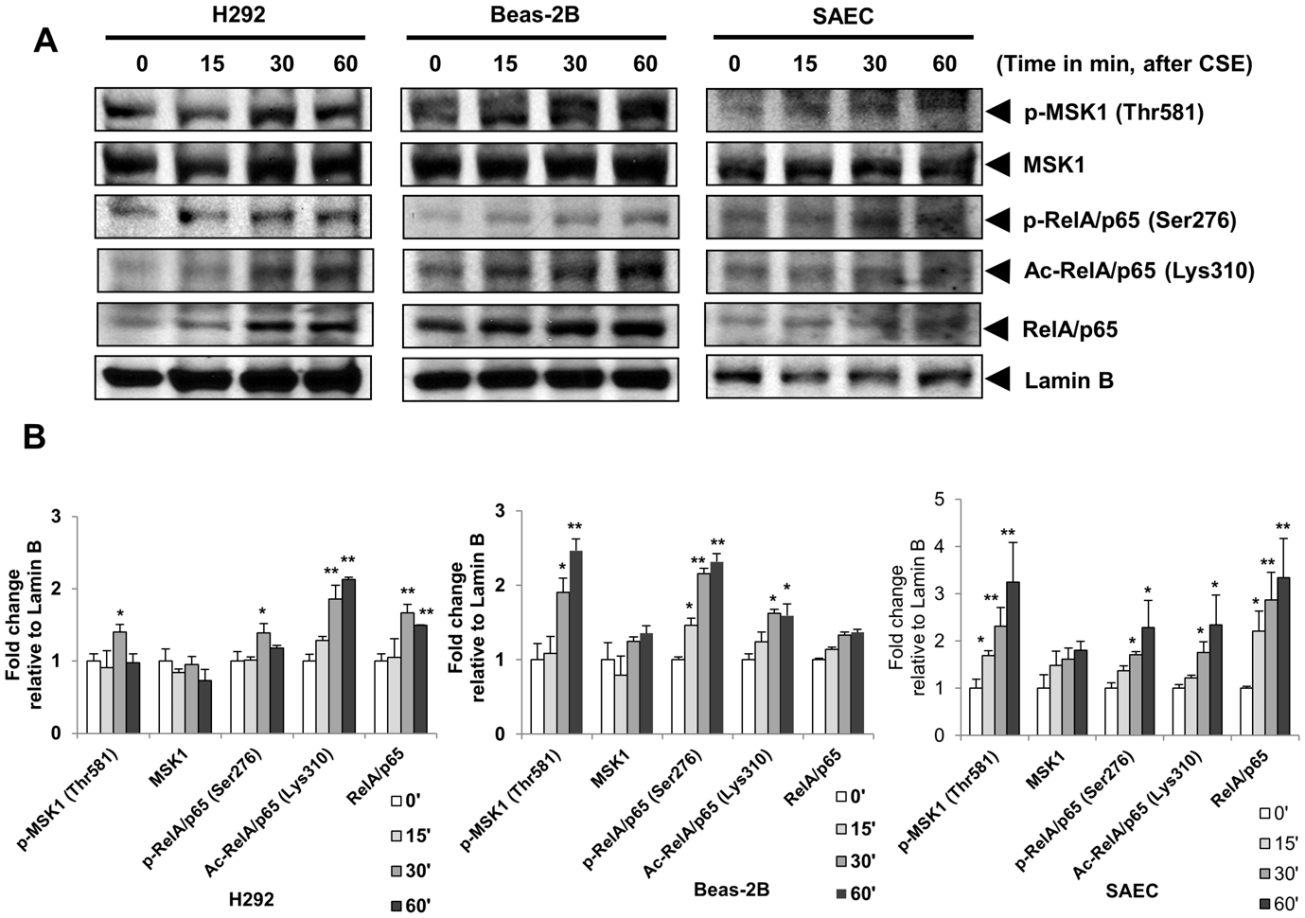
Cigarette smoke increased the nuclear levels of phosphorylated MSK1, phosphorylated and acetylated RelA/p65 in a time-dependent manner in human bronchial and small airway epithelial cells, and in mouse lung. (A) H292, BEAS-2B and SAEC cells were treated with or without 1% CSE (time course: 0, 15, 30, and 60 minutes). Cells were harvested, nuclear extracts were prepared and immunoblotted for p-MSK1 (Thr581), total MSK1, phosphorylated RelA/p65 (Ser276), acetylated RelA/p65 (Lys310), and total RelA/p65. Lamin B was used as nuclear protein loading controls. Gel pictures shown are representative of at least three separate experiments. (B) The band intensity was measured by densitometry and data shown as fold change relative to Lamin B control. Data are shown as mean ± SEM; *, *P*<0.05; **, *P*<0.01 significant compared with control or without treatment.

**Figure 2:**
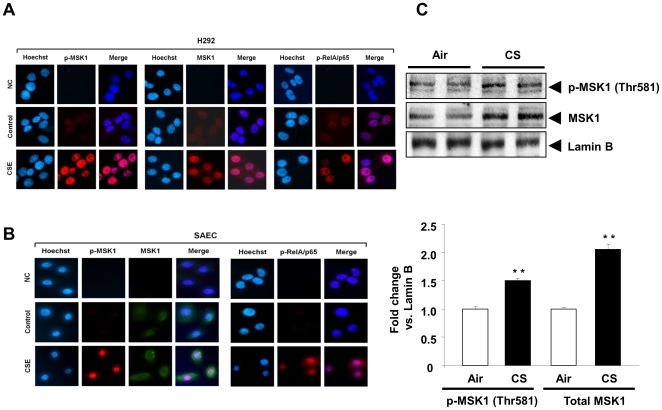
Cigarette smoke increased p-MSK1 (Thr581) and p-RelA/p65 (Ser276) in human bronchial and small airway epithelial cells as demonstrated by immunocytochemistry, and in mouse lung by immunoblotting. (A) Activation of MSK1 and RelA/p65 (Ser276) in H292 cells treated with CSE (1%) for 1 h. For immunocytochemistry, cells were fixed, permeabilized, and stained for the expression of p-MSK1 (Thr581), total MSK1, and phosphorylated RelA/p65 (Ser276) by immunofluorescence. Phosphorylated MSK1, total MSK1, and phosphorylated RelA/p65 is shown in red, and DNA (Hoechst nuclear staining) in blue, and merge represented as dark purple or pink. Results are representative cells from three separate experiments. The panel without primary antibodies was considered as negative control (NC). (B) Activation of MSK1 and RelA/p65 (Ser276) in SAEC cells treated with CSE (1%) for 1 h. For immunocytochemistry, cells were fixed, permeabilized, and stained for the expression of p-MSK1 (Thr581), total MSK1, and phosphorylated RelA/p65 (Ser276) by immunofluorescence. Phosphorylated MSK1, and phosphorylated RelA/p65 is shown in red, total MSK1 in green, and DNA (Hoechst nuclear staining) in blue, and merge represented as dark purple or pink. Results are representative cells from three separate experiments. The panel without primary antibodies was considered as negative control (NC). (C) Nuclear levels of MSK1 (phosphorylated and total MSK1) were increased in lung tissue of mouse exposed to CS determined by immunoblot analysis. Adult C57BL/6J mice were exposed to CS for 3 days and were sacrificed 24 hrs post-final CS exposure. Lamin B was used as protein loading control. The band intensity was measured by densitometry and data shown as fold change relative to Lamin B control. Data are shown as mean ± SEM (n = 4/group); **, *P*<0.01, significant compared to air-exposed controls. p-MSK1 (Thr581), phosphorylated MSK1.

MSK1 is activated by both mitogens and stress in different physiological and pathological conditions [26]. We hypothesized that CS activates MSK1, leading to phospho-acetylation of the RelA/p65 subunit of NF-κB in human bronchial epithelial cells (H292 and BEAS-2B), and human primary small airway epithelial cells (SAEC). Human epithelial cells were treated with different doses of CSE (0.1% to 1.0%) at different time points (0 min–60 min). Cells were harvested, nuclear extracts prepared, and immunoblot analysis performed to examine CS-induced activation of nuclear MSK1 (MSK1 phosphorylation at Thr581) and phospho-acetylation of RelA/p65 at Ser276 and Lys310. Interestingly, nuclear accumulation of phosphorylated MSK1 (Thr581), and p-RelA/p65 (Ser276), and levels of Ac-RelA/p65 (Lys310) were increased in a time-dependent manner in H292, BEAS-2B and SAEC cells, (Fig. 1A–B), as well as in mouse lung (Fig. 2C). To further investigate CS-mediated activation of MSK1, immunocytochemistry was performed in H292 and SAEC cells after 1 h CSE (1%) treatment, using antibodies specific for p-MSK1 (Thr581), total MSK1 and p-RelA/p65 (Ser276), and immunohistochemistry for MSK1 in CS-exposed mouse lung sections. CSE treatment resulted in increased nuclear levels of p-MSK1, MSK1 and p-RelA/p65 (Ser276) compared to non-treated controls (Fig. 2A–B). Immunohistochemistry of mouse lung sections also revealed a significant increase in the levels of MSK1 in alveolar epithelium, alveolar type II cells, and airway epithelium of CS-exposed mouse lung compared to air-exposed controls (data not shown). These findings confirm that CS activates MSK1 and its substrate causing phospho-acetylation of NF-κB RelA/p65 in human transformed epithelial cells, primary small airway epithelial cells, and mouse lung.

### CSE-mediated activation of MSK1 is associated with phospho-acetylation of histone H3 and acetylation of histone H4

**Figure 3:**
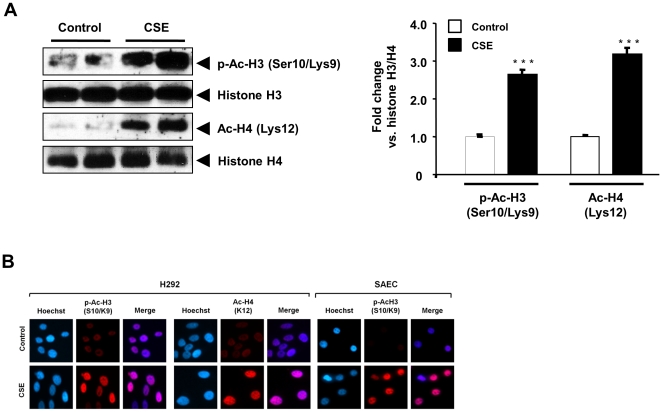
CSE increased phospho-acetylation of histone H3 (Ser10/Lys9) and acetylation of H4 (Lys12) in H292 cells. Acid extracted histone proteins were used for immmunoblotting against anti-phospho-acetylated histone H3 (Ser10/Lys9) and acetylated histone H4 (Lys12). (A) The levels of phospho-acetylated (histone H3 on Ser10/Lys9) and acetylated (histone H4 on Lys12) histones were increased in response to CSE in H292 cells. The band intensity was measured by densitometry and data shown as fold change relative to total histones H3/H4. Data are shown as mean ± SEM (n = 3). ***, *P*<0.001 significant compared to control group. p-Ac-H3, phospho-acetylated histone H3; Ac-H4, acetylated histone H4. (B) Immunocytochemistry showing histone modifications in H292 and SAEC cells treated with CSE (1%) for 1 h. For immunocytochemistry, cells were fixed, permeabilized, and stained for expression of phospho-acetylated histone H3 (Ser10/Lys9) and acetylated histone H4 (Lys12) by immunofluorescence. Phospho-acetylated histone H3 and acetylated histone H4 are shown in red, and DNA (Hoechst nuclear staining) in blue, and merge represented as dark purple or pink. Gel pictures and immunocytochemistry figures are representative of at least three separate experiments.

Phosphorylation of histone H3 at Ser10 and acetylation of histone H4 on Lys12 have been shown to occur early during chromatin remodeling [10], [27]. To elucidate the downstream signaling events involved in chromatin modifications in response to CSE treatment in H292 cells, we performed immunoblot analysis to determine the levels of Ser10 phosphorylation and Lys9 acetylation of histone H3 and Lys12 acetylation of histone H4 in acid-extracted histones. Phospho-acetylation of histone H3 and acetylation of histone H4 were increased in response to CSE treatment in H292 cells (Fig. 3A). Additionally, immunocytochemistry for p-Ac-H3 (Ser10/Lys9), and Ac-H4 (Lys12) was performed using specific antibodies in H292 and SAEC cells treated for 1 h with CSE (1%). CSE caused phospho-acetylation of histone H3 and acetylation of histone H4 in H292 and SAEC cells (Fig. 3B), suggesting that CS/acrolein-mediated activation of MSK1 further caused histone modifications.

### Knock-down of MSK1 inhibits CSE-induced RelA/p65 activation and histone modifications

In order to gain mechanistic insight into MSK1-mediated activation of RelA/p65 and posttranslational histone modifications, we determined the downstream events i.e. the phosphorylation of RelA/p65 (Ser276) and histone modifications in response to CSE using a MSK1 N- and C-terminal kinase-dead mutant and siRNA-mediated knock-down approaches, as well as using a stable MSK1 knock-down mouse embryonic fibroblasts (MEFs).

**Figure 4:**
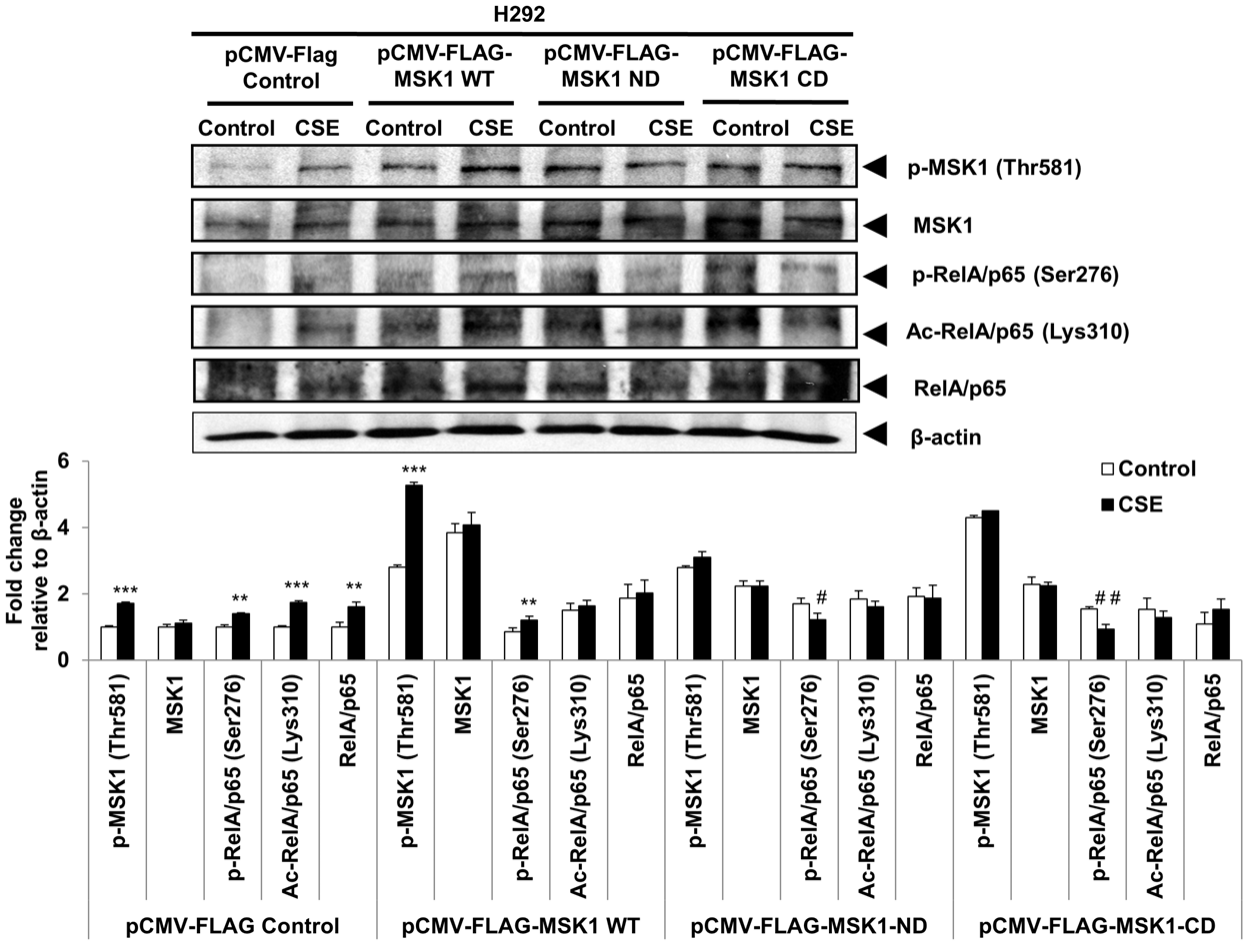
MSK1 N- and C-terminal kinase-dead mutants in H292 cells attenuated the phosphorylation of RelA/p65 by CSE treatment. Cigarette smoke increased the cellular levels of phosphorylated MSK1 as well as phosphorylated RelA/p65 levels in control vector (pCMV-FLAG) and MSK1 WT (pCMV-FLAG-MSK1 wild-type) transfected human bronchial epithelial cells compared to MSK1 N- and C-terminal kinase-dead mutants (pCMV-FLAG-MSK1 ND and pCMV-FLAG-MSK1 CD). H292 cells were transiently transfected with control vector, MSK1 WT, MSK1 N-terminal kinase-dead (ND) mutant, MSK1 C-terminal kinase-dead (CD) mutant and treated with or without CSE (1%) after 24-48 hrs of transfection for 1 h. Cells were harvested (1 h later). Whole cell extracts were prepared and immunoblotted for p-MSK1 (Thr581), total MSK1, phosphorylated RelA/p65 (Ser276), acetylated RelA/p65 (Lys310), and total RelA/p65. β-actin was used as protein loading control. Gel pictures are representative of at least three separate experiments. The band intensity was measured by densitometry and data shown as fold change relative to β-actin control. Data are shown as mean ± SEM; **, *P*<0.01; ***, *P*<0.001, significant compared with respective control group; #, *P*<0.05; ##, *P*<0.01; significant compared to CSE treated control vector transfected group.

First, we transiently transfected H292 cells with control vector (pCMV-FLAG), an expression vector for MSK1wild-type (pCMV-FLAG-MSK1 WT), MSK1 N-terminal kinase-dead (ND) mutant (pCMV-FLAG-MSK1 D195A), and MSK1 C-terminal kinase-dead (CD) mutant (pCMV-FLAG-MSK1 D565A). CSE-mediated activation of MSK1 (p-MSK1) and phosphorylation of RelA/p65 were significantly increased in H292 cells transfected with control vector and MSK1 wild-type. CS-induced phosphorylation of MSK1 and RelA/p65 were modest but significantly decreased without any change in acetylation of RelA/p65 in both MSK1 N- and C-terminal kinase-dead mutant transfected H292 cells (Fig. 4). Our results suggest that both N-terminal and C-terminal kinase domains are crucial for MSK1 activation in human bronchial epithelial cells.

**Figure 5:**
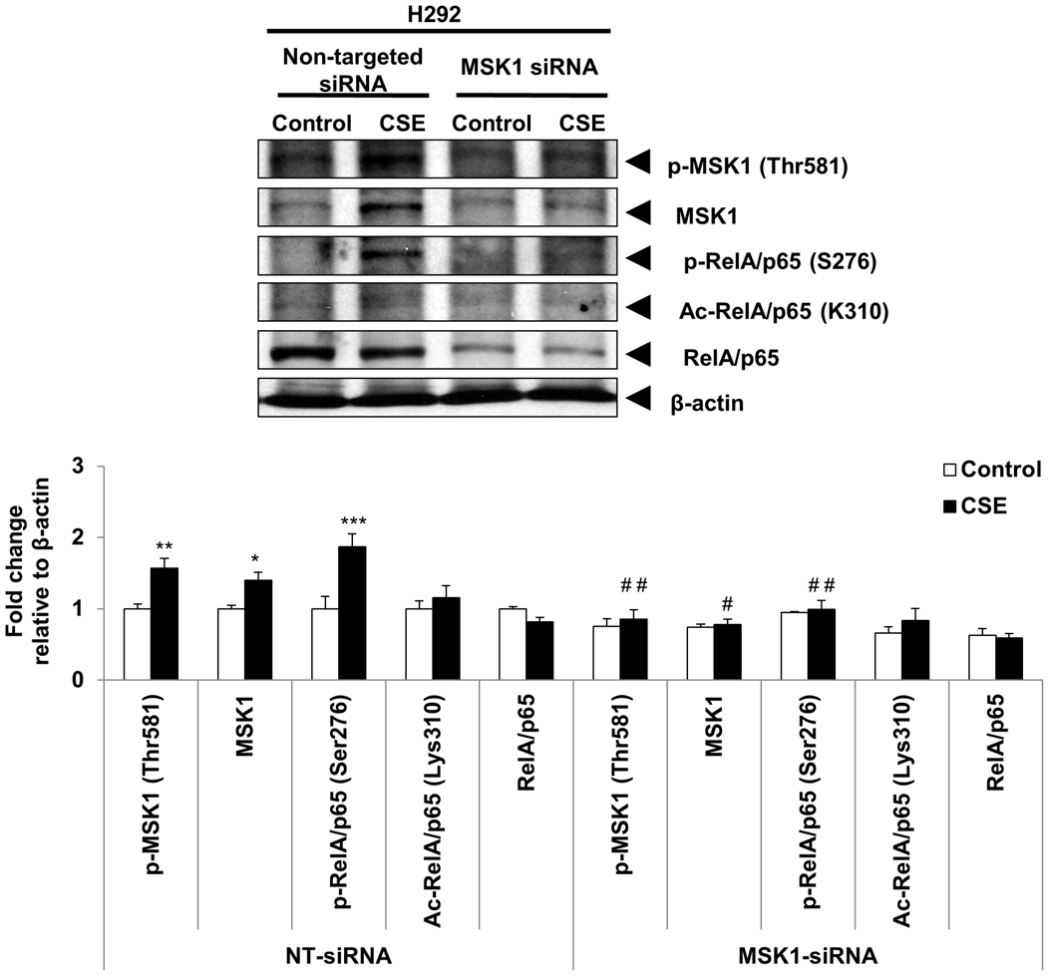
siRNA-mediated knock-down of MSK1 in H292 cells attenuated the phosphorylation of RelA/p65 by CSE treatment. MSK1 knock-down by siRNA approach attenuated the phosphorylation of RelA/p65 by CSE in human bronchial epithelial cells. H292 cells were transfected with 100 nM scrambled siRNA (non-targeted siRNA) or siRNA directed against MSK1 (MSK1 siRNA) for 24 h, followed by starvation in serum free medium and treated for 1 h after 72 h of transfection with CSE (1%). Whole cell lysate was prepared after 1 h treatment and assayed for effect of MSK1 knock-down by immunoblotting. The levels of p-MSK1 (Thr581), total MSK1, phosphorylated RelA/p65 (Ser276), acetylated RelA/p65 (Lys310), and total RelA/p65 were determined by immunoblotting. β-actin was used as a loading control. Gel pictures are representative of at least three separate experiments. The band intensity was measured by densitometry and data shown as fold change relative to β-actin control. Data are shown as mean ± SEM; *, *P*<0.05; **, *P*<0.01; ***, *P*<0.001, significant compared with control or without treatment non-targeted siRNA group; #, *P*<0.05; ##, *P*<0.01, significant compared to CSE treated non-targeted siRNA group.

**Figure 6:**
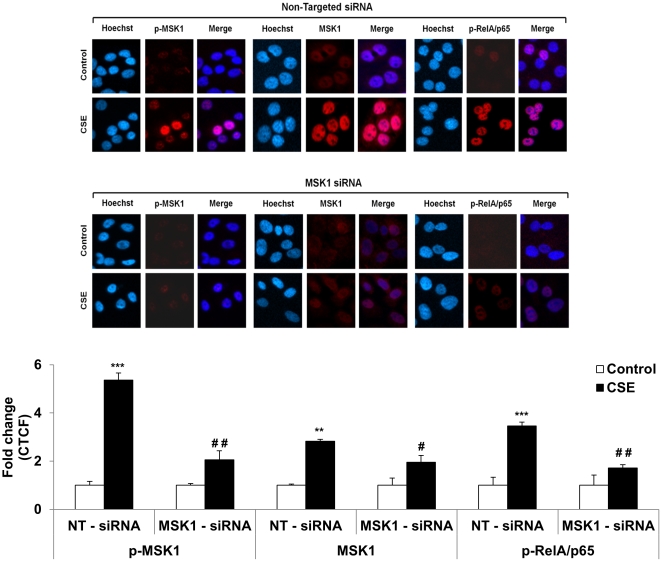
MSK1 knock-down by MSK1 siRNA in H292 cells attenuated the phosphorylation of RelA/p65 by CSE treatment demonstrated by immunocytochemistry. CSE-induced activation of MSK1 and RelA/p65 (Ser276) in non-targeted siRNA transfected human bronchial epithelial cells. MSK1 knock-down by siRNA approach attenuated the activation of MSK1 and phosphorylation of RelA/p65 by CSE in human bronchial epithelial cells. H292 cells were transfected with 100 nM scrambled siRNA (non-targeted siRNA) or MSK1 siRNA for 24 h, followed by starvation in serum free medium and then treated for 1 h (72 h post-transfection) with CSE (1.0%). For immunocytochemistry, cells were fixed, permeabilized, and stained for the expression of p-MSK1 (Thr581), total MSK1, and phosphorylated RelA/p65 (Ser276) by immunofluorescence. Phosphorylated MSK1, total MSK1, and phosphorylated RelA/p65 are shown in red, and DNA (Hoechst nuclear staining) in blue, and merge represented as dark purple or pink. Results are representative cells from at least three separate experiments. The quantification of fluorescence intensity in immunofluorescence data was measured using ImageJ and the corrected total cell fluorescence (CTCF) values were converted into fold change values and represented as histograms. Data are shown as mean ± SEM; **, *P*<0.01; ***, *P*<0.001, significant compared with respective control without treatment; #, *P*<0.05; ##, *P*<0.01, significant compared to CSE treated non-targeted siRNA group.

Our second approach to examine the effect of decreased MSK1 activity involved transient siRNA-mediated knockdown of MSK1 expression. H292 cells were transiently transfected with MSK1 target-specific siRNA or with non-targeted control siRNA. MSK1 siRNA transfection resulted in a reduction in endogenous MSK1 levels of more than 70%. This reduction was associated with a modest but significant reduction in the levels of p-MSK1 (Thr581), total MSK1, and phosphorylated RelA/p65 (Ser276) compared to non-targeted control siRNA transfected cells (Fig. 5). Similarly, immunocytochemistry confirmed this phenomenon when H292 cells were transiently transfected with MSK1 siRNA and treated with CSE by showing a significant reduction in the levels of p-MSK1 and phosphorylated RelA/p65 (Ser276) compared to non-targeted siRNA transfected human bronchial epithelial cells (Fig. 6A–B).

**Figure 7:**
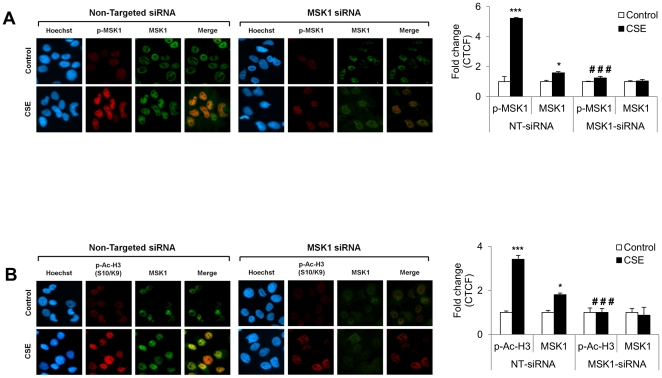
MSK1 knock-down by MSK1 siRNA in H292 cells attenuated the phospho-acetylation of histone H3/H4 by CSE treatment demonstrated by immunocytochemistry. (A) MSK1 knock-down by siRNA approach attenuated activation of MSK1 by CSE in H292 cells. CS-induced activation of MSK1 (p-MSK1 Thr581) was observed in control siRNA transfected cells compared to MSK1 siRNA transfected human bronchial epithelial cells. H292 cells were transfected with 100 nM scrambled siRNA (non-targeted siRNA) or siRNA directed against MSK1 (MSK1 siRNA) for 24 h, followed by starvation in serum free medium and treated for 1 h after 72 h of transfection with CSE (1.0%). For immunocytochemistry, cells were fixed, permeabilized, and stained for the expression of p-MSK1 (Thr581), and total MSK1 by immunofluorescence. Phospho-MSK1 is shown in red, and total MSK1 is shown green, and DNA (Hoechst nuclear staining) in blue, and merge represented as orange yellow. (B) MSK1 knock-down by siRNA approach attenuated CSE-induced histone modifications (phospho-acetylated histone H3) in human bronchial epithelial cells. H292 cells were transfected with 100 nM scrambled siRNA (non-targeted siRNA) or siRNA directed against MSK1 (MSK1 siRNA) for 24 h, followed by starvation in serum free medium and treated for 1 h after 72 h of transfection with CSE (1.0%). For immunocytochemistry, cells were fixed, permeabilized, and stained for the expression of p-Ac-H3 (Ser10/Lys9), and total MSK1 by immunofluorescence. Phospho-acetylated histone H3 is shown in red, and total MSK1 is shown green, and DNA (Hoechst nuclear staining) in blue, and merge represented as orange yellow. Results are representative cells from at least three separate experiments. p-Ac-H3, phospho-acetylated histone H3. Quantification of fluorescence intensity in immunofluorescence data was measured using ImageJ and the corrected total cell fluorescence (CTCF) values were converted into fold change values and represented as histograms. Data are shown as mean ± SEM; *, *P*<0.05; ***, *P*<0.001, significant compared with respective control or without treatment; ###, *P*<0.001, significant compared to CSE treated non-targeted siRNA group.

**Figure 8:**
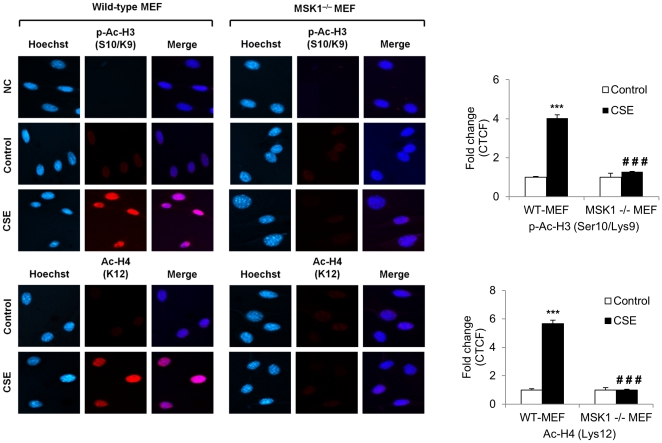
MSK1 knock-down using MSK1 stable knock-down MEFs attenuated the phospho-acetylation of histone H3/H4 by CSE treatment demonstrated by immunocytochemistry. CSE induced histone modification (phosphorylated/acetylated histone H3/H4) in 10T1/2 wild-type mouse embryonic fibroblasts (MEFs) compared to stable MSK1 knock-down MEFs. Wild-type MEFs and MSK1 knock-down MEFs were treated for 1 h with CSE (1.0%). For immunocytochemistry, cells were fixed, permeabilized and stained for the expression of p-Ac H3 (Ser10/Lys9), and Ac-H4 (Lys12) by immunofluorescence. Phosphorylated/acetylated histone H3 and acetylated histone H4 are shown in red, and DNA (Hoechst nuclear staining) in blue, and merge represented as purple or pink. Results are representative cells from at least three separate experiments. The panel without primary antibodies was considered as negative control (NC). The quantification of fluorescence intensity in immunofluorescence data was measured using ImageJ and the corrected total cell fluorescence (CTCF) values were converted into fold change values and represented as histograms. Data are shown as mean ± SEM; ***, *P*<0.001, significant compared with control or without treatment; ###, *P*<0.001, significant compared to CSE treated wild-type group.

We next determined the effect of MSK1 siRNA-mediated knockdown on activation of p-MSK1 (Thr581), and chromatin modifications [phospho-acetylation of histone H3 (Ser10/Lys9)] in H292 cells. Immunocytochemistry was performed in transfected cells treated with CSE (1%) for 72 hr to measure p-MSK1, total MSK1, and phospho-acetylation of histone H3 (Ser10/Lys9) using specific antibodies. Knock-down of MSK1 using MSK1 siRNA led to a significant decrease in phosphorylated and total levels of MSK1, as well as a decrease in phospho-acetylation of histone H3 (Ser10/Lys9) in response to CSE treatment compared to non-targeted control siRNA-transfected H292 cells (Fig. 7A–B). In addition, the reduction in phospho-acetylation of histone H3 (Ser10/Lys9) and acetylation of histone H4 (Lys12) was also evident in MSK1 stable knock-down mouse embryonic fibroblast (MEF) cells treated with CSE compared to wild-type MEFs (10T1/2 cells) as shown by immunocytochemistry (Fig. 8). These data suggest that activation of MSK1 is a key event in response to CS leading to NF-κB activation and histone modifications.

### CS-mediated interaction of MSK1 with its substrate RelA/p65 and coactivator p300

**Figure 9:**
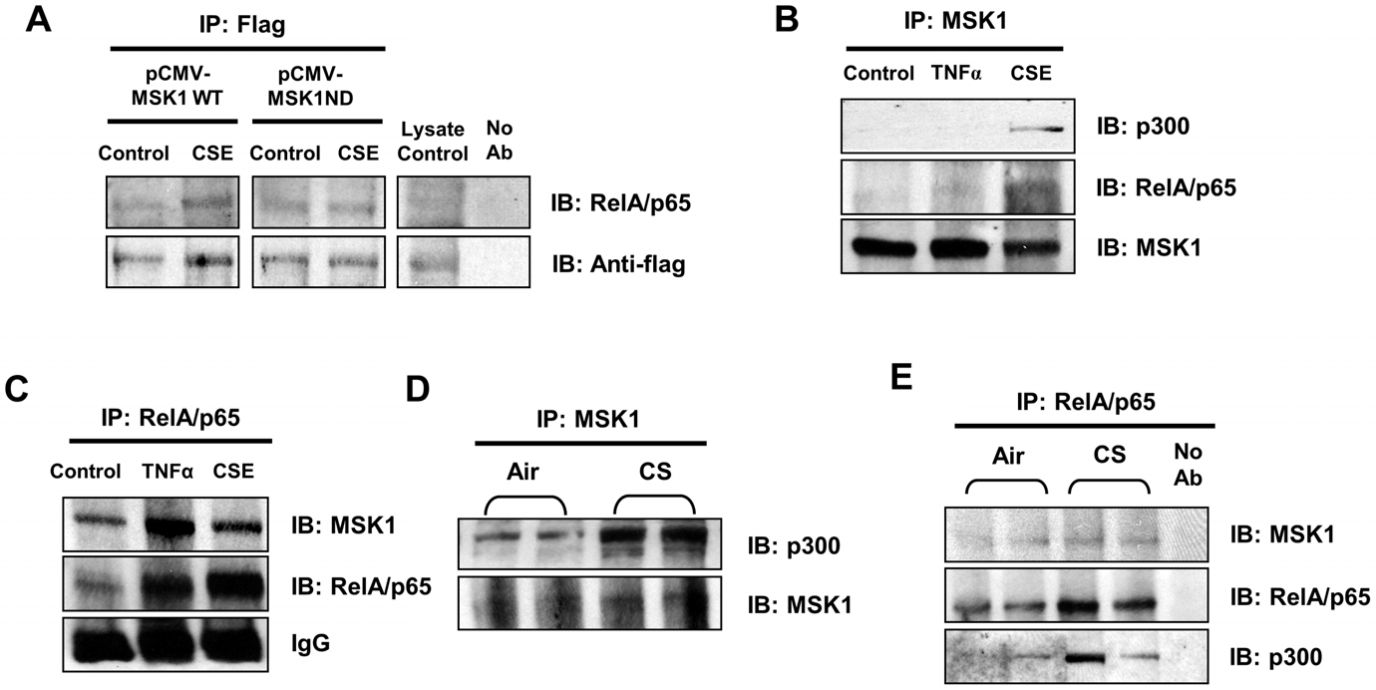
MSK1 interacted with RelA/p65 and p300. (A) H292 cells were transiently transfected with wild-type MSK1 or MSK1 N-terminal kinase-dead mutant MSK1 flag construct and treated with or without CSE (1.0%) for 1 h. Flag-tagged precipitated complexes were analyzed through immunoblotting using anti-flag and anti-RelA/p65 antibodies. Whole cell lysate from pCMV-Flag-MSK1 wild-type transfected cells were used as lysate control. (B) H292 cells were treated for 1 h with or without CSE (2.0%) and TNFα (10 ng/ml) was used as positive control. Nuclear extract (1000 µg) was used for immunoprecipitation of MSK1 bound complexes and analyzed through immunoblotting using anti-MSK1, anti-RelA/p65 and anti-p300 antibodies. (C) H292 cells were treated for 1 h with or without CSE (2.0%), and TNFα (10 ng/ml) was used as positive control. Nuclear extract (1000 µg) was used for immunoprecipitation of RelA/p65 bound complexes and analyzed through immunoblotting using anti-RelA/p65 and anti-MSK1 antibodies. (D) CS-mediated interaction of MSK1-p300 in acute CS exposed mouse lung. Adult C57BL/6J mice were exposed to CS at 300 mg/m^3^ total particulate matter (TPM) for 3 days and were sacrificed 24 h after the final exposure. Whole tissue extract (500 µg) was used for immunoprecipitation of MSK1 bound complexes, and analyzed by immunoblotting using anti-MSK1 and anti-p300 antibodies. (E) CS-mediated interaction of RelA/p65 with MSK1 and p300 in acute CS exposed mouse lung. Adult C57BL/6J mice were exposed to CS at 300 mg/m^3^ total particulate matter (TPM) for 3 days and were sacrificed 24 h after the final exposure. Whole tissue extract (500 µg) was used for immunoprecipitation of RelA/p65 bound complexes, and analyzed by immunoblotting using anti-RelA/p65, anti-MSK1 and anti-p300 antibodies. Results are representative of at least three separate experiments.

Knock-down of MSK1 significantly reduced the MSK1-mediated downstream signaling events activated by CS. We, therefore, speculated that MSK1 directly interacts with and activates RelA/p65 early during chromatin modifications, and thus also with cofactors of RelA/p65. We performed immunoprecipitations in H292 cells treated with or without CSE (1% for 1 hr), which were transiently transfected with MSK1 wild-type (pCMV-FLAG-MSK1-WT) or with MSK1 N-terminal kinase-dead mutant (pCMV-FLAG-MSK1-ND). Whole cell lysates were prepared to isolate MSK1 complexes by anti-flag immunoprecipitation, and analyzed by immunoblotting for co-immunoprecipitation of MSK1 with RelA/p65. CSE-mediated interaction of MSK1 and RelA/p65 was evident in pCMV-FLAG-MSK1 wild-type transfected cells, but the ratio of RelA/p65 and MSK1 was not significantly changed (Fig. 9A). This may be due to the relative increased levels of MSK1 and RelA/p65 pool in the cells treated with CSE compared to that in control cells. Interaction between MSK1 and RelA/p65 in CSE-treated pCMV-FLAG-MSK1-ND transfected cells was lower compared to pCMV-FLAG-MSK1 WT transfected cells (Fig. 9A). Based on this finding and the observation on involvement of both N-terminal and C-terminal kinase domains in MSK1 activation (Fig. 4), we expect similar effects on MSK1 interaction with RelA/p65 after transfection of pCMV-FLAG-MSK1 CD in H292 cells and treatment with CSE. To examine the association of native MSK1 with RelA/p65, and with cofactor p300, we treated H292 cells with CSE (2%) for 1 h and performed immunoprecipitation from nuclear extracts using anti-MSK1 and anti-RelA/p65 antibodies. Interaction of MSK1 with RelA/p65 and with the cofactor p300 was evident in response to CSE treatment as compared to control in H292 cells (Fig. 9B–C). These findings suggest that the interactions between MSK1, RelA/p65, and p300 occur by CSE.

To demonstrate the interplay between RelA/p65-MSK1, MSK1-p300, and RelA/p65-MSK1-p300 *in vivo*, lung homogenates from air- and CS-exposed mouse lungs were used for immunoprecipitation using anti-RelA/p65 and anti-MSK1 antibodies. RelA/p65 complexes isolated by anti-RelA/p65 immunoprecipitations were analyzed by immunoblotting so as to determine the interaction of MSK1 with RelA/p65, MSK1 with p300, and RelA/p65 with p300. Interaction between RelA/p65-MSK1, MSK1-p300, and RelA/p65-p300 was significantly increased in CS-exposed mouse lung compared to air-exposed control (Fig. 9D–E). This confirms the interplay between RelA/p65-MSK1, MSK1-p300, as well as RelA/p65-MSK1-p300, and activation and interaction of these complexes both *in vitro* in H292 cells and *in vivo* in mouse lungs is mediated by CS. These interactions may play a key role in chromatin remodeling on the promoters of pro-inflammatory genes.

### CSE treatment causes the recruitment of MSK1 along with phorphorylated RelA/p65 and acetylated histone H3 and histone H4 on the promoters of NF-κB-dependent pro-inflammatory genes

**Figure 10:**
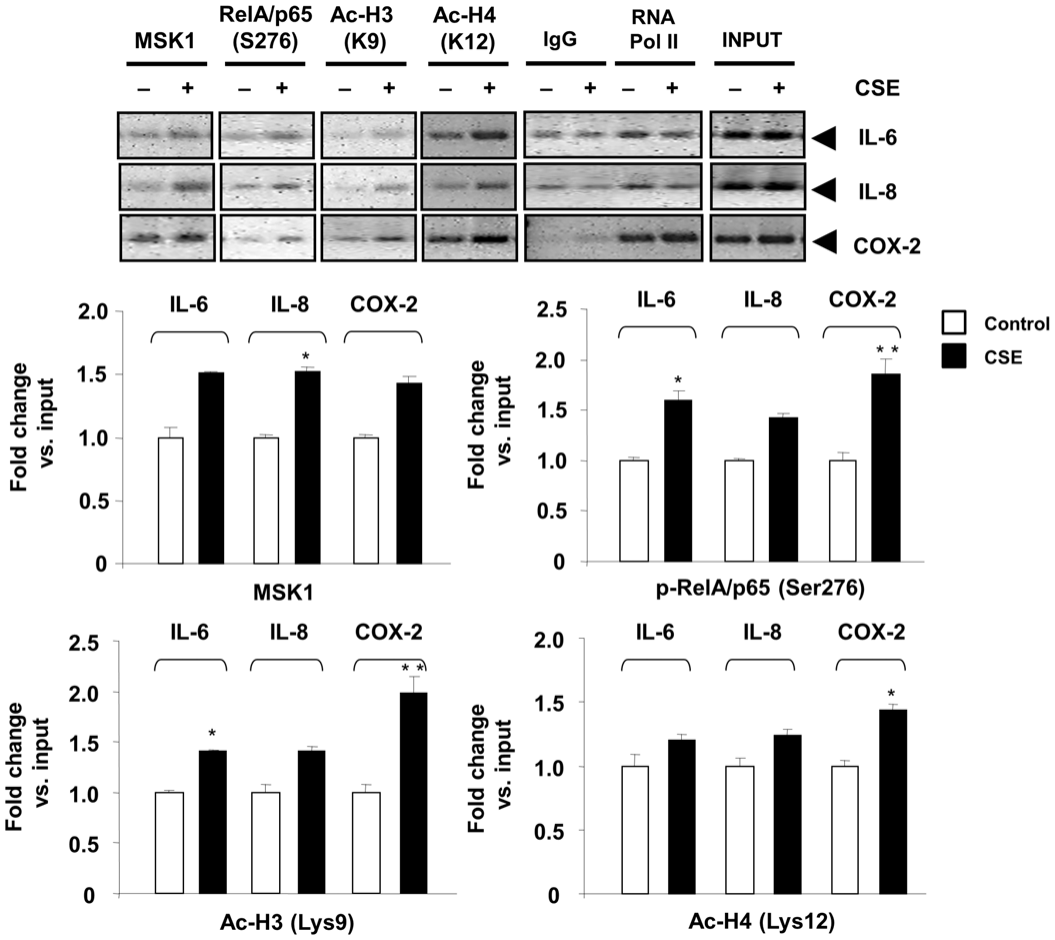
ChIP assay revealed that MSK1 is localized to pro-inflammatory gene promoters (IL-8, IL-6 and COX-2). H292 cells were treated with CSE (1%) for 1 h. ChIP analysis was performed against MSK1, phospho-RelA/p65 (Ser276), phospho-histone H3 (Lys9) and acetylated histone H4 (Lys12) antibodies. After reverse cross-linking, co-immunoprecipitated genomic DNA fragments were analyzed by semi-quantitative PCR with IL-6, IL-8, and COX-2 promoter specific primer sets. IgG was used as negative control, and RNA Polymerase II antibody was used as positive control. Input reflects the relative amount of sonicated DNA fragments present before immunoprecipitation and revealed by semi-quantitative PCR with pro-inflammatory gene specific primers. The band intensity was measured by densitometry and data shown as fold change versus input. Data are shown as mean ± SEM (n = 3). *, *P*<0.05; **, *P*<0.01, significant compared to respective control group.

We further demonstrated the role of MSK1 on NF-κB-driven pro-inflammatory genes using the chromatin immunoprecipitation (ChIP) assay *in vitro* (H292 cells). CS-induced nuclear activation of MSK1 resulted in its localization to NF-κB-dependent pro-inflammatory gene promoters, including IL-6, IL-8, and COX-2. The ChIP assay revealed that upon CSE treatment, MSK1, phosphorylated RelA/p65, acetylated histone H3 (Lys9), and histone H4 (Lys12) were localized to these pro-inflammatory gene promoters to induce gene transcription (Fig. 10). These data suggest that CS-induced MSK1 recruitment on the promoters of pro-inflammatory genes drives their transcription via RelA/p65 phosphorylation and histone acetylation. Overall, these data establish the fact that MSK1 not only phosphorylates transcription factor NF-κB RelA/p65 and causes chromatin modifications (phospho-acetylated histone H3 and acetylated histone H4), but also results in recruitment of MSK1 and its substrates on NF-κB-responsive pro-inflammatory gene promoters culminating in chromatin modifications and gene transcription in response to CS.

## Discussion

Cigarette smoke is the common etiological factor in the pathogenesis of COPD. We and others have demonstrated the role of the NF-κB signaling pathway in pro-inflammatory gene transcription and chromatin modifications [1], [2], [10], [28], [29], [30]. Studies in the past have shown that NF-κB is recruited to the promoter of pro-inflammatory genes in CS-exposed rodent lungs, resulting in increased histone acetylation [10], [31], [32], [33].

MSK1 has been shown to regulate transcription of several pro-inflammatory genes at multiple levels [34]. Transcription factor CREB was the first MSK1 substrate to be identified [17], [35]. MSK1 also phosphorylates RelA/p65 at Ser276 residue in response to TNFα [9]. In addition to transcription factor regulation, MSK1 can contribute to gene activation by phosphorylation of chromatin proteins histone H3 (Ser10 and Ser28) and HMG-N1 (Ser6) [11]. However, the underlying molecular signaling mechanism that causes activation of MSK1 and downstream signaling event, including chromatin histone modifications, in response to CS is not known. We tested the hypothesis that CS induces histone modifications via activation of MSK1 and phospho-acetylation of RelA/p65 followed by recruitment of MSK1 and its substrates to the promoters of pro-inflammatory genes, thereby increasing the transcriptional activity of pro-inflammatory mediators in human bronchial and small airway epithelial cells and in mouse lungs.

CS exposure resulted in phosphorylation of MSK1 at Thr581 in transformed human bronchial epithelial cells (H292 and BEAS-2B), human primary small airway epithelial cells (SAEC) and in mouse lung. We observed similar dose-dependent activation of MSK1 and RelA/p65 by CSE (0.1% to 1.0%) in H292, BEAS-2B and SAEC cells (data not shown). Phosphorylation of MSK1 at Thr581 residue is an event of activated MSK1 protein [13], [36]. MSKs are activated by several mitogenic and stress stimuli, such as epidermal growth factor (EGF), phorbol 12-myristate 13-acetate/TPA and UV possibly via oxidative stress [17], [37], [38]. Our results show that CS activates MSK1, leading to phospho-acetylation of RelA/p65 at Ser276 and Lys310, respectively, in epithelial cells. Similar observations were found when the cells were treated with a component of CS, the aldehyde acrolein (data not shown). Other studies have shown the control of phosphorylation of RelA/p65 at multiple serine residues regulates transcriptional activity of NF-κB RelA/p65 by TNF-α, IL-1β, UV, farnesol, respiratory syncytial virus (RSV) in various cell types [39], [40], [41], [42], [43]. Hence, it is possible that MSK1 activation regulates RelA/p65 phosphorylation in lung epithelial cells in response to CS. This is shown by the evidence that CS causes a significant increase in the levels of phosphorylated MSK1 and phospho-acetylation of RelA/p65 in control vector and wild-type MSK1 transfected H292 cells. There was a modest but significant reduction of MSK1 activation and RelA/p65 phosphorylation without any change in acetylation of RelA/p65 in MSK1 N- and C-terminal kinase-dead mutant transfected H292 cells suggest the role of both N- and C- terminal domains of MSK1 are crucial for it activation. Based on previous report [17], we speculated that the kinase activity will be affected in cells transfected with MSK1 N- and C-terminal kinase-dead mutants without any appreciable effects on MSK1 levels. Nevertheless, CS-induced activation of MSK1 and RelA/p65 was modest, but a significant reduction was seen in MSK1 siRNA transfected H292 cells compared to non-targeted control siRNA transfected cells. This suggests that the activation of RelA/p65 (Ser276) is MSK1-dependent in epithelial cells.

MSK1 contains two catalytic active kinase domains (N- and C-terminal), which are required for proper function [13], [17]. Earlier reports show that neither of the kinase-dead mutants (N- or C-terminal) of MSK1 possessed detectable activity nor showed any change in the total levels of MSK1 either before or after stimulation of cells with TPA or exposure to UV [17], [42], [44], [45]. Therefore, both the N- and C-terminal kinase domains play an essential role in activity of MSKs [11], [42], [44]. MSK1 also acts as a regulator of inflammation [46], [47]. However, the signaling mechanism by which CS activates MSK1 is not known. It has been shown that IKKα translocated into the nucleus and is required for optimal NF-κB-mediated transcription and phosphorylation of histone H3 at Ser10 of NF-κB target genes [10], [28], [48], [49], [50], as well as EGF-induced transcriptional regulation of immediate early genes (IEGs) [51], [52]. Hence, we proposed that CS activates MSK1 via IKKα, leading to chromatin modifications in human lung epithelial cells. In support of this, we found that CS increases the levels of phosphorylated MSK1 and phospho-acetylated RelA/p65 in H292 cells overexpressing IKKα compared both to cells expressing a dominant-negative IKKα and to non-transfected control cells. These data suggest that IKKα plays an important role in CSE-induced activation of MSK1 and histone modifications in epithelial cells. Immunocytochemistry data also suggest that CS-mediated activation of MSK1 occurs via IKKα (unpublished observations). Recently, we have reported that NF-κB inducing kinase (NIK) activation by CS and TNFα induces RelA/p65 and histone H3K9 acetylation in human bronchial epithelial cells. Nuclear accumulation and recruitment of NIK on the proinflammatory gene promoters result in NF-κB-dependent gene activation [53].

Our data show that CS-mediated activation of MSK1 further leads to phospho-acetylation of histone H3 (Ser10/Lys9) and acetylation of histone H4 (Lys12) in bronchial epithelial cells. These modifications were confirmed by LC-MS/MS analysis of histone H3 and H4 fractions from CS-treated H292 cells. Apart from this, MS analysis also revealed CSE-mediated phosphorylation of histone H3 at Ser28 and acetylation of histone H4 at Lys5, Lys8, Lys12 and Lys16 (unpublished observations). Our *in vitro* data are in agreement with other studies on MSK1-mediated histone H3 Ser10 and Ser28 phosphorylation [11], [54], [55], [56]. Earlier reports have shown that IKKα phosphorylates histone H3 Ser10, and RelA/p65 phospho-acetylation (Ser276/Lys310) occurs by direct interaction with CBP/p300 [10], [30], [48], [49]. We further demonstrate that CS-mediated activation of MSK1 leads to phospho-acetylation of histone H3 (Ser10/Lys9), which was significantly reduced when MSK1 was knocked down in human bronchial epithelial cells, as well as in stable MSK1 knock-down mouse embryonic fibroblast (MEF) treated with CSE. Similarly, histone H3 is phosphorylated at Ser10 or Ser28 in response to other mitogenic or stress stimuli associated with induction of IEGs [16], [44], [57]. Notably, studies using MSK1 and MSK2 knock-out MEFs or using the MSK1 dominant-negative mutant or MSK1 knock-down approaches have demonstrated that these kinases play a vital role in phosphorylation of histone H3 Ser10 and Ser28 in various cell lines [9], [11], [54], [55], [58], [59], [60]. Thus, our data highlight the importance of MSK1-mediated nucleosomal response by CS-mediated oxidative/carbonyl stress potentially leading to inflammatory response.

We have observed CS-induced direct interactions between MSK1 and RelA/p65 in epithelial cells *in vitro* and in acute CS-exposed mouse lung *in vivo* by coimmunoprecipitation. p300 also coimmunoprecipitated with MSK1 and RelA/p65 suggesting that acetylation of histones H3 and H4 in response to CS is mediated by CBP/p300. This interaction plays a vital role in the regulation of DNA binding by transcription factors, such as NF-κB to the promoters of pro-inflammatory genes [61]. Recruitment of MSK1 along with other DNA-binding proteins and coactivators, such as CBP/p300 may result in phosphorylation of chromatin proteins, particularly histone H3 (Ser10 or Ser28), and thus activate gene transcription [62]. MSK1 interaction with coactivators (CBP/p300 and ER81) and other chromatin modifying enzymes stimulate the transactivation of target genes [16], [45], [60]. Earlier reports have described that phosphorylation of RelA/p65 at Ser276 enhances its assembly with CBP/p300 [14], [39], [40], and this interaction is augmented due to the acetylation of a key lysine residues in RelA/p65 (Lys310), which potentiates transcriptional activity [7], [63]. Similarly, we have previously reported that CS/aldehyde- or LPS-induced lung inflammation resulted in acetylation of RelA/p65 and histone modifications (phospho-acetylation of histone H3) via the interaction of RelA/p65 with coactivator CBP/p300 in mouse lung [30], [53]. Thus, based on our data, we conclude that CS/aldehyde-induced MSK1 activation leads to the formation of a complex that includes MSK1, RelA/p65 and CBP/p300, which localizes to pro-inflammatory gene promoters and modifies histones to promote transcriptional activation.

We also speculated that MSK1-mediated phosphorylation of RelA/p65 at Ser276 is required for the RelA/p65 and p300 in response to CSE. In support of this, we found that a S276A mutant Flag-tagged RelA/p65 protein did not coimmunoprecipitate with p300 either in the presence or absence of CSE (unpublished observations). This suggests a crucial role for RelA/p65 phosphorylation as an important event in CS-mediated MSK1 activation and NF-κB signaling. Our ChIP analysis revealed that MSK1, and its substrates RelA/p65 (Ser276), acetylated histone H3 (Lys9) and histone H4 (Lys12) are recruited to the promoters of pro-inflammatory genes in response to CS in epithelial cells. This is similar to the findings that CS causes recruitment of IKKα and RelA/p65 to the promoters of pro-inflammatory genes, such as MIP-2 and IL-6 in mouse lung [10], [31]. Earlier study by Gilmour *et al.* demonstrated a role of histone H4 acetylation in regulation of IL-8 gene expression using the ChIP assay showing an increased association of acetylated H4 on IL-8 gene promoter following TSA, PM10, and TNF treatments after 24 h [64]. In light of this, we propose a similar phenomenon that CSE treatment in H292 cells for 24 hrs may show a dynamic recruitment of MSK1, phosphorylated RelA/p65, and acetylated histone H3 and H4 on the promoters of pro-inflammatory genes. Thus, MSK1 kinase appears not only to modify and activate the factors involved in transcriptional regulation, but also participate in the complex that mediates chromatin remodeling on pro-inflammatory gene promoters. This is corroborated by the findings of Beck *et al.* who demonstrated the presence of MSK1 on inflammatory gene promoters, proximal to or in the κB-site, which was significantly reduced by glucocorticoids in A549 epithelial cells [43]. Similarly, other studies have demonstrated that MSK1 and its substrates RelA/p65 (Ser276) and phospho-histone H3 (Ser10) were localized to pro-inflammatory promoters [9], [65], [66]. Thus, MSK1 plays an active role in linking the signaling cascade and gene transcription in response to pro-inflammatory environmental stimuli, such as CS and aldehydes.

**Figure 11:**
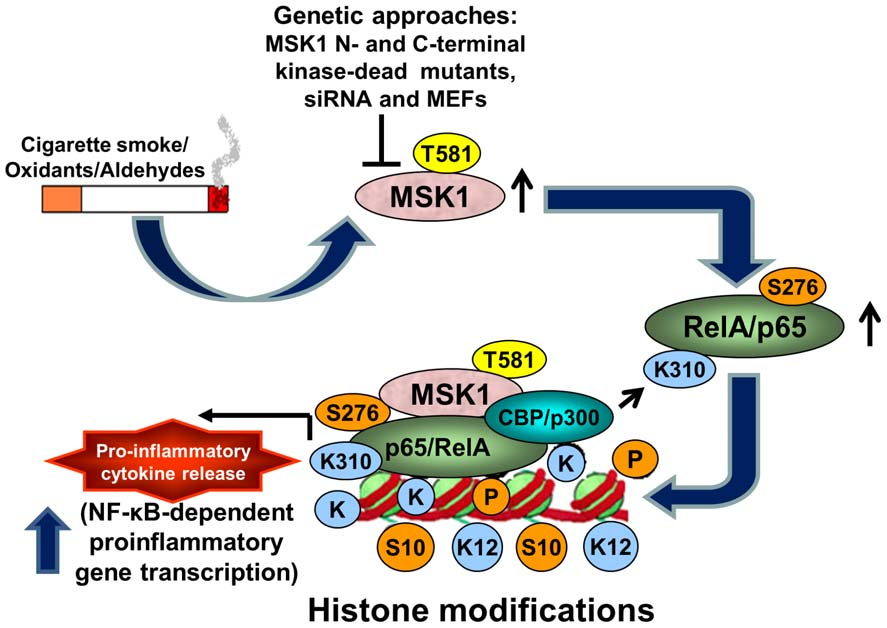
A schematic model showing the role of MSK1 in cigarette smoke-induced NF-κB signaling and chromatin modifications. Cigarette smoke/aldehyde/oxidative stress via activation of MSK1 results in phosphorylation, and nuclear transclocation of NF-κB RelA/p65. Activated nuclear MSK1 subsequently causes chromatin modifications of histones (phospho-acetylation of histone H3 [Ser10/Lys9] and acetylation of histone H4 [Lys12]). MSK1 N-terminal kinase dead mutant, siRNA-mediated knock-down and stable MSK1 knock-down MEFs represses the nuclear MSK1, phosphorylation of RelA/p65, and phospho-acetylation of histone H3 and acetylation of histone H4. CS activates MSK1 which forms a complex with NF-κB RelA/p65 and coactivator p300 which further mediates transcriptional activation of NF-κB-dependent pro-inflammatory genes (IL-6, IL-8 and COX-2). (↑, Induction; ⊢ inhibition).

In summary, we have demonstrated a novel role of MSK1 in CS-induced activation of NF-κB RelA/p65 (Ser276/Lys310) and chromatin modifications in human lung epithelial cells and mouse lung (Fig. 11). MSK1 serves as a specific NF-κB RelA/p65 kinase, promoting transcriptional activation of RelA/p65-dependent pro-inflammatory genes via IKKα-mediated activation of MSK1 and RelA/p65. Knock-down of MSK1 reduces CSE-induced activation of MSK1, RelA/p65 phosphorylation and posttranslational modifications of histones. CS-induced interactions between MSK1, RelA/p65 and p300 play a crucial role in sustained pro-inflammatory gene transcription by causing acetylation of RelA/p65 at Lys310, and modulating chromatin modifications at specific histone residues both *in vitro* and *in vivo*. CSE-induced MSK1-mediated phosphorylation of RelA/p65 at Ser276 is required for the interaction of RelA/p65 with p300. The ChIP assay demonstrates that MSK1 and its substrates associate with the promoters of NF-κB-dependent pro-inflammatory genes. These findings provide direct evidence that MSK1 is a kinase that plays a crucial role in CS-induced chromatin modifications. Thus, MSK1 represents a potential target for therapy in controlling CS-mediated chronic inflammatory response seen in several diseases, including COPD and lung cancer.

## References

[1] Rahman I, Adcock IM (2006). Oxidative stress and redox regulation of lung inflammation in COPD.. Eur Respir J.

[2] Rajendrasozhan S, Chung S, Sundar IK, Yao H, Rahman I (2010). Targeted disruption of NF-{kappa}B1 (p50) augments cigarette smoke-induced lung inflammation and emphysema in mice: a critical role of p50 in chromatin remodeling.. Am J Physiol Lung Cell Mol Physiol.

[3] Rajendrasozhan S, Yao H, Rahman I (2009). Current perspectives on role of chromatin modifications and deacetylases in lung inflammation in COPD.. COPD.

[4] Barnes PJ, Shapiro SD, Pauwels RA (2003). Chronic obstructive pulmonary disease: molecular and cellular mechanisms.. Eur Respir J.

[5] Yao H, Edirisinghe I, Rajendrasozhan S, Yang SR, Caito S (2008). Cigarette smoke-mediated inflammatory and oxidative responses are strain-dependent in mice.. Am J Physiol Lung Cell Mol Physiol.

[6] Kode A, Yang SR, Rahman I (2006). Differential effects of cigarette smoke on oxidative stress and proinflammatory cytokine release in primary human airway epithelial cells and in a variety of transformed alveolar epithelial cells.. Respir Res.

[7] Chen LF, Williams SA, Mu Y, Nakano H, Duerr JM (2005). NF-kappaB RelA phosphorylation regulates RelA acetylation.. Mol Cell Biol.

[8] Yang SR, Wright J, Bauter M, Seweryniak K, Kode A (2007). Sirtuin regulates cigarette smoke-induced proinflammatory mediator release via RelA/p65 NF-kappaB in macrophages in vitro and in rat lungs in vivo: implications for chronic inflammation and aging.. Am J Physiol Lung Cell Mol Physiol.

[9] Vermeulen L, De Wilde G, Van Damme P, Vanden Berghe W, Haegeman G (2003). Transcriptional activation of the NF-kappaB p65 subunit by mitogen- and stress-activated protein kinase-1 (MSK1).. EMBO J.

[10] Yang SR, Valvo S, Yao H, Kode A, Rajendrasozhan S (2008). IKK alpha causes chromatin modification on pro-inflammatory genes by cigarette smoke in mouse lung.. Am J Respir Cell Mol Biol.

[11] Soloaga A, Thomson S, Wiggin GR, Rampersaud N, Dyson MH (2003). MSK2 and MSK1 mediate the mitogen- and stress-induced phosphorylation of histone H3 and HMG-14.. EMBO J.

[12] Kefaloyianni E, Gaitanaki C, Beis I (2006). ERK1/2 and p38-MAPK signalling pathways, through MSK1, are involved in NF-kappaB transactivation during oxidative stress in skeletal myoblasts.. Cell Signal.

[13] McCoy CE, Campbell DG, Deak M, Bloomberg GB, Arthur JS (2005). MSK1 activity is controlled by multiple phosphorylation sites.. Biochem J.

[14] Zhong H, Voll RE, Ghosh S (1998). Phosphorylation of NF-kappa B p65 by PKA stimulates transcriptional activity by promoting a novel bivalent interaction with the coactivator CBP/p300.. Mol Cell.

[15] Reddel RR, Ke Y, Gerwin BI, McMenamin MG, Lechner JF (1988). Transformation of human bronchial epithelial cells by infection with SV40 or adenovirus-12 SV40 hybrid virus, or transfection via strontium phosphate coprecipitation with a plasmid containing SV40 early region genes.. Cancer Research.

[16] Drobic B, Perez-Cadahia B, Yu J, Kung SK, Davie JR (2010). Promoter chromatin remodeling of immediate-early genes is mediated through H3 phosphorylation at either serine 28 or 10 by the MSK1 multi-protein complex.. Nucleic Acids Res.

[17] Deak M, Clifton AD, Lucocq LM, Alessi DR (1998). Mitogen- and stress-activated protein kinase-1 (MSK1) is directly activated by MAPK and SAPK2/p38, and may mediate activation of CREB.. EMBO J.

[18] Kim YM, Cao D, Reed W, Wu W, Jaspers I (2007). Zn2+-induced NF-kappaB-dependent transcriptional activity involves site-specific p65/RelA phosphorylation.. Cell Signal.

[19] Kode A, Rajendrasozhan S, Caito S, Yang SR, Megson IL (2008). Resveratrol induces glutathione synthesis by activation of Nrf2 and protects against cigarette smoke-mediated oxidative stress in human lung epithelial cells.. Am J Physiol Lung Cell Mol Physiol.

[20] Carp H, Janoff A (1978). Possible mechanisms of emphysema in smokers. In vitro suppression of serum elastase-inhibitory capacity by fresh cigarette smoke and its prevention by antioxidants.. Am Rev Respir Dis.

[21] Fujioka K, Shibamoto T (2006). Determination of toxic carbonyl compounds in cigarette smoke.. Environ Toxicol.

[22] Lambert C, McCue J, Portas M, Ouyang Y, Li J (2005). Acrolein in cigarette smoke inhibits T-cell responses.. J Allergy Clin Immunol.

[23] Carnevali S, Nakamura Y, Mio T, Liu X, Takigawa K (1998). Cigarette smoke extract inhibits fibroblast-mediated collagen gel contraction.. Am J Physiol.

[24] Smith CJ, Hansch C (2000). The relative toxicity of compounds in mainstream cigarette smoke condensate.. Food Chem Toxicol.

[25] Mauderly JL, Gigliotti AP, Barr EB, Bechtold WE, Belinsky SA (2004). Chronic inhalation exposure to mainstream cigarette smoke increases lung and nasal tumor incidence in rats.. Toxicol Sci.

[26] Arthur JS (2008). MSK activation and physiological roles.. Front Biosci.

[27] Strahl BD, Allis CD (2000). The language of covalent histone modifications.. Nature.

[28] Yamamoto Y, Verma UN, Prajapati S, Kwak YT, Gaynor RB (2003). Histone H3 phosphorylation by IKK-alpha is critical for cytokine-induced gene expression.. Nature.

[29] Ito K, Charron CE, Adcock IM (2007). Impact of protein acetylation in inflammatory lung diseases.. Pharmacol Ther.

[30] Yao H, Hwang JW, Moscat J, Diaz-Meco MT, Leitges M (2010). Protein kinase C zeta mediates cigarette smoke/aldehyde- and lipopolysaccharide-induced lung inflammation and histone modifications.. J Biol Chem.

[31] Yang SR, Yao H, Rajendrasozhan S, Chung S, Edirisinghe I (2009). RelB is differentially regulated by IkappaB Kinase-alpha in B cells and mouse lung by cigarette smoke.. Am J Respir Cell Mol Biol.

[32] Marwick JA, Kirkham PA, Stevenson CS, Danahay H, Giddings J (2004). Cigarette smoke alters chromatin remodeling and induces proinflammatory genes in rat lungs.. Am J Respir Cell Mol Biol.

[33] Yang SR, Chida AS, Bauter MR, Shafiq N, Seweryniak K (2006). Cigarette smoke induces proinflammatory cytokine release by activation of NF-kappaB and posttranslational modifications of histone deacetylase in macrophages.. Am J Physiol Lung Cell Mol Physiol.

[34] Vermeulen L, Vanden Berghe W, Beck IM, De Bosscher K, Haegeman G (2009). The versatile role of MSKs in transcriptional regulation.. Trends Biochem Sci.

[35] Wiggin GR, Soloaga A, Foster JM, Murray-Tait V, Cohen P (2002). MSK1 and MSK2 are required for the mitogen- and stress-induced phosphorylation of CREB and ATF1 in fibroblasts.. Mol Cell Biol.

[36] Markou T, Lazou A (2002). Phosphorylation and activation of mitogen- and stress-activated protein kinase-1 in adult rat cardiac myocytes by G-protein-coupled receptor agonists requires both extracellular-signal-regulated kinase and p38 mitogen-activated protein kinase.. Biochem J.

[37] Pierrat B, Correia JS, Mary JL, Tomas-Zuber M, Lesslauer W (1998). RSK-B, a novel ribosomal S6 kinase family member, is a CREB kinase under dominant control of p38alpha mitogen-activated protein kinase (p38alphaMAPK).. J Biol Chem.

[38] New L, Zhao M, Li Y, Bassett WW, Feng Y (1999). Cloning and characterization of RLPK, a novel RSK-related protein kinase.. J Biol Chem.

[39] Jamaluddin M, Tian B, Boldogh I, Garofalo RP, Brasier AR (2009). Respiratory syncytial virus infection induces a reactive oxygen species-MSK1-phospho-Ser-276 RelA pathway required for cytokine expression.. J Virol.

[40] Reber L, Vermeulen L, Haegeman G, Frossard N (2009). Ser276 phosphorylation of NF-kB p65 by MSK1 controls SCF expression in inflammation.. PLoS One.

[41] Joo JH, Jetten AM (2008). NF-kappaB-dependent transcriptional activation in lung carcinoma cells by farnesol involves p65/RelA(Ser276) phosphorylation via the MEK-MSK1 signaling pathway.. J Biol Chem.

[42] Jacks KA, Koch CA (2010). Differential regulation of mitogen- and stress-activated protein kinase-1 and -2 (MSK1 and MSK2) by CK2 following UV radiation.. J Biol Chem.

[43] Beck IM, Vanden Berghe W, Vermeulen L, Bougarne N, Vander Cruyssen B (2008). Altered subcellular distribution of MSK1 induced by glucocorticoids contributes to NF-kappaB inhibition.. EMBO J.

[44] Zhong S, Jansen C, She QB, Goto H, Inagaki M (2001). Ultraviolet B-induced phosphorylation of histone H3 at serine 28 is mediated by MSK1.. J Biol Chem.

[45] Perez-Cadahia B, Drobic B, Espino PS, He S, Mandal S (2011). Role of MSK1 in the malignant phenotype of Ras-transformed mouse fibroblasts.. J Biol Chem.

[46] Ananieva O, Darragh J, Johansen C, Carr JM, McIlrath J (2008). The kinases MSK1 and MSK2 act as negative regulators of Toll-like receptor signaling.. Nat Immunol.

[47] Darragh J, Ananieva O, Courtney A, Elcombe S, Arthur JS (2010). MSK1 regulates the transcription of IL-1ra in response to TLR activation in macrophages.. Biochem J.

[48] Anest V, Hanson JL, Cogswell PC, Steinbrecher KA, Strahl BD (2003). A nucleosomal function for IkappaB kinase-alpha in NF-kappaB-dependent gene expression.. Nature.

[49] Gloire G, Horion J, El Mjiyad N, Bex F, Chariot A (2007). Promoter-dependent effect of IKKalpha on NF-kappaB/p65 DNA binding.. J Biol Chem.

[50] Chung S, Sundar IK, Yao H, Ho YS, Rahman I (2010). Glutaredoxin 1 regulates cigarette smoke-mediated lung inflammation through differential modulation of I{kappa}B kinases in mice: impact on histone acetylation.. Am J Physiol Lung Cell Mol Physiol.

[51] Anest V, Cogswell PC, Baldwin AS (2004). IkappaB kinase alpha and p65/RelA contribute to optimal epidermal growth factor-induced c-fos gene expression independent of IkappaBalpha degradation.. J Biol Chem.

[52] Duncan EA, Anest V, Cogswell P, Baldwin AS (2006). The kinases MSK1 and MSK2 are required for epidermal growth factor-induced, but not tumor necrosis factor-induced, histone H3 Ser10 phosphorylation.. J Biol Chem.

[53] Chung S, Sundar IK, Hwang JW, Yull FE, Blackwell TS (2011). NF-kappaB Inducing Kinase, NIK Mediates Cigarette Smoke/TNFalpha-Induced Histone Acetylation and Inflammation through Differential Activation of IKKs.. PLoS One.

[54] Chadee DN, Hendzel MJ, Tylipski CP, Allis CD, Bazett-Jones DP (1999). Increased Ser-10 phosphorylation of histone H3 in mitogen-stimulated and oncogene-transformed mouse fibroblasts.. J Biol Chem.

[55] Dunn KL, Davie JR (2005). Stimulation of the Ras-MAPK pathway leads to independent phosphorylation of histone H3 on serine 10 and 28.. Oncogene.

[56] Garcia BA, Barber CM, Hake SB, Ptak C, Turner FB (2005). Modifications of human histone H3 variants during mitosis.. Biochemistry.

[57] Thomson S, Clayton AL, Hazzalin CA, Rose S, Barratt MJ (1999). The nucleosomal response associated with immediate-early gene induction is mediated via alternative MAP kinase cascades: MSK1 as a potential histone H3/HMG-14 kinase.. EMBO J.

[58] Kannan-Thulasiraman P, Katsoulidis E, Tallman MS, Arthur JS, Platanias LC (2006). Activation of the mitogen- and stress-activated kinase 1 by arsenic trioxide.. J Biol Chem.

[59] Kim HG, Lee KW, Cho YY, Kang NJ, Oh SM (2008). Mitogen- and stress-activated kinase 1-mediated histone H3 phosphorylation is crucial for cell transformation.. Cancer Res.

[60] Janknecht R (2003). Regulation of the ER81 transcription factor and its coactivators by mitogen- and stress-activated protein kinase 1 (MSK1).. Oncogene.

[61] Chen L, Fischle W, Verdin E, Greene WC (2001). Duration of nuclear NF-kappaB action regulated by reversible acetylation.. Science.

[62] Berger SL (2002). Histone modifications in transcriptional regulation.. Curr Opin Genet Dev.

[63] Chen LF, Mu Y, Greene WC (2002). Acetylation of RelA at discrete sites regulates distinct nuclear functions of NF-kappaB.. EMBO J.

[64] Gilmour PS, Rahman I, Donaldson K, MacNee W (2003). Histone acetylation regulates epithelial IL-8 release mediated by oxidative stress from environmental particles.. Am J Physiol Lung Cell Mol Physiol.

[65] Okazaki T, Sakon S, Sasazuki T, Sakurai H, Doi T (2003). Phosphorylation of serine 276 is essential for p65 NF-kappaB subunit-dependent cellular responses.. Biochem Biophys Res Commun.

[66] Caivano M, Cohen P (2000). Role of mitogen-activated protein kinase cascades in mediating lipopolysaccharide-stimulated induction of cyclooxygenase-2 and IL-1 beta in RAW264 macrophages.. J Immunol.

